# Nanostructured Oxides Obtained by Anodizing Aluminum Intermetallic Alloys

**DOI:** 10.3390/ma18225192

**Published:** 2025-11-15

**Authors:** Paulina Chilimoniuk-Szwarc, Piotr Dobroń, Wojciech Jerzy Stępniowski

**Affiliations:** Institute of Materials Science and Engineering, Faculty of Advanced Technologies and Chemistry, Military University of Technology, 2 Kaliskiego Street, 00908 Warsaw, Poland; paulina.chilimoniuk@wat.edu.pl (P.C.-S.); piotr.dobron@student.wat.edu.pl (P.D.)

**Keywords:** anodizing, aluminum alloys, nanopores, nanostructures, anodic oxidation, iron aluminides, nickel aluminides, titanium aluminides, purple gold

## Abstract

Aluminum anodizing has been a well-established method of corrosion protection for over a century. A nanoporous and hexagonally arranged anodic aluminum oxide has become one of the most important template materials in nanotechnology. A totally new branch of research in anodizing was sparked by purple gold anodizing. This pioneering research showed that metal aluminides can be anodized and result in new classes of nanomaterials. Simultaneously, materials from Ti-Al systems were anodized, and the transition from nanopores to the nanotubes was mechanistically understood. Also, materials like Ni_3_Al were anodized; however, the most frequently used aluminides are materials from the Fe-Al binary phase diagram, from Fe_3_Al to FeAl_3_. The research on metal aluminides has shown that it is possible to obtain mixed oxides with a highly developed nanostructured morphology. A significant amount of fundamental research has shown it is possible to obtain such mixed oxides with tunable band gaps, depending on the substrate material, anodizing conditions, and heat treatment. Despite significant progress in fundamental research, there is a noticeable lack of applied research on this class of materials.

## 1. Introduction

Anodizing is an electrochemical method of passive layer formation on metals by oxidation. Originally, it has been used for over a century to protect technical aluminum alloys from corrosion [1]. Currently, the most modern trends in technical aluminum alloys anodizing focus on pulse anodizing [2,3,4], searching for Cr(VI) substitutes [5,6,7,8], or covering the porous oxide with corrosion inhibitors, i.e., via the sol–gel technique [9,10]. Another trend explored for three decades is the formation of highly ordered, nanoporous anodic aluminum oxide (AAO), made of honey-comb-like, hexagonally arranged nanopores. The inventors, Masuda and Fukuda, obtained for the first time, thanks to the two-step self-organized anodizing, perfectly organized honey-comb-like nanopores [11]. With this approach, one of the most important template materials, anodic aluminum oxide, was obtained in a facile and reproductible way. Later, the scientists discovered that the morphological features, like pore diameter, interpore distance, or thickness of the oxide layer can be controlled by the operating conditions, including type, concentration and temperature of the electrolyte, anodizing voltage, duration of the process, or even by viscosity of the electrolyte [12,13,14,15,16,17]. Also, perfection of the nanopores arrangement was found to be impacted by the operating conditions; thus, it was found that only for certain operating conditions set in certain electrolytes, highly ordered nanopores can be obtained [18,19,20]. For example, an almost perfect, honey-comb-like hexagonal ordering of the pores grown on pure aluminum is obtained in 0.3 M oxalic acid at voltages ranging from 40 to 50 V [18,19]. It has provided much fundamental knowledge and also allowed to steer the geometry of the template material. Since then, via various methods, nanowires [21,22,23,24,25,26,27,28,29,30], nanotubes [30,31,32], and nanodots [33] made of metals [21,22,23,24,25,33], oxides [26,27,30,31,32], or polymers [28,29] were reported to be obtained. Such nanomaterials have found numerous applications in sensing [26,34,35,36], catalysis [23,37,38,39,40], renewable energy harvesting and storage [41], or microelectronics [24,42].

Self-organized anodization was expanded into other metals, like Ti [43,44,45,46,47,48,49], Ta [50,51], Nb [52], Zr [53], Sn [54], W [55], Zn [56,57], or Cu [58,59,60,61]. Nanostructured oxides formed by the self-organized anodizing allowed to contribute in such emerging applications like assembly of photocatalysis [45,48,55], dye sensitized solar cells [46,47], green hydrogen generation [62], electrochemical carbon dioxide reduction reaction [59], micro and nanoplastic removal [60], or methanol oxidation [61].

There is also another trend—anodization of aluminum alloys with a large content of alloying elements. It combines nanoporous morphology acknowledged to aluminum with semiconductive properties of the second element. Such an approach opens numerous opportunities in applications, like photocatalysis, providing a developed surface area of a semiconductive oxide. The main intention of the paper is to systematize the anodizing protocols for the anodization of aluminum alloys with a significant amount of alloying elements and combine them with properties and resulting applications. Aluminum intermetallic anodizing is of particular scientific interest for a couple of reasons: simultaneous oxidation of aluminum and a second (i.e., Au, Ni, Fe) metal, both present in the unit cell of an alloy, can provide the formation of nanostructured morphology of an oxide. Such an oxide will contain both elements in the form of cations, which allows one to tailor the composition of the nanostructured oxide. Furthermore, what is known from the anodization of pure elements, operating conditions can allow the design of the morphology of the nanoporous/nanotubular product of anodizing. Anodization of metal aluminides also allows control of physical properties of the formed oxides, like band gap, which can be beneficial in further applications. This review, as the first, intends to gather and systemize the information about findings related to intermetallic alloy anodizing.

## 2. Anodization of Purple Gold

Historically, one of the first aluminides anodized was AuAl_2_, also known as purple gold. In the Au-Al binary phase diagram, there are five intermetallic phases, namely AuAl_2_, AuAl, Au_2_Al, Au_5_Al_2_, and Au_4_Al, but due to the highest Al:Au ratio, only AuAl_2_ was subjected to anodizing. Nishio et al. reported purple gold anodizing in oxalic acid (0.3 M oxalic acid, 5, or 20 V, 30 or 90 min; Table 1) [63]. Typically, anodization of aluminum results in the formation of a hexagonally arranged AAO [11,12,13], while in the case of purple gold, nanoparticles with an average diameter of ca. 30 nm were obtained. The longer the anodization time, the greater the surface coverage with the nanoparticles. It has been proven that the obtained nanoparticles are pure gold; therefore, selective oxidation of aluminum in the AuAl_2_ took place. The Authors say that the clear mechanism is still unknown, but re-arrangement of Au atoms on the surface, forming the nanoparticles, has to be one of the steps. It also means that aluminum was selectively oxidized, while Au was kept at the Au^0^ oxidation state. When bare “single-atoms” of gold were exposed on the surface, these had to organize in greater clusters (the nanoparticles) to lower the surface energy. Such nanoparticles, at first glance, provided a surface color change. Such a change indicated the surface plasmon resonance in the visible range. This means that the obtained Au nanoparticles strongly absorb and enhance electromagnetic fields when illuminated.

Additionally, the spectral characterization of the surface differed after anodizing due to the localized surface plasmons in the Au nanoparticles. Pyridine molecules were attached to the surfaces before and after anodizing, and it was shown that the anodized purple gold with the Au nanoparticles is an efficient Raman substrate (Surface-Enhanced Raman Spectroscopy; SERS). The formed Au nanoparticles locally increase the intensity of the electromagnetic field, thus the intensity of specific bands of pyridine was significantly amplified.

An interesting fact is that later, the same group, anodized pure gold, also in oxalic acid, and nanoporous, sponge-like gold oxide, was obtained [64]. It means that both pure elements provide nanoporous oxide by anodizing, in the same electrolyte, oxalic acid, but intermetallic alloy anodizing provides nanoparticles, which makes anodization of intermetallic alloys unique.

## 3. Anodization of Ti-Al Alloys

**Figure 1:**
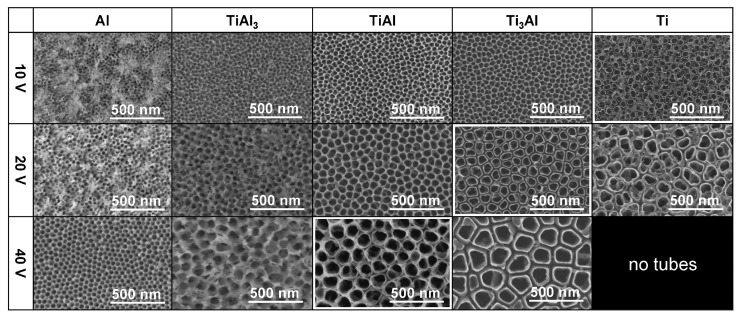
Top-view FE-SEM images of Al, Ti, and their intermetallics anodized in 1 M H_2_SO_4_ containing 0.15 wt.% HF at various voltages. Reprinted with permission from ACS [65].

**Figure 2:**
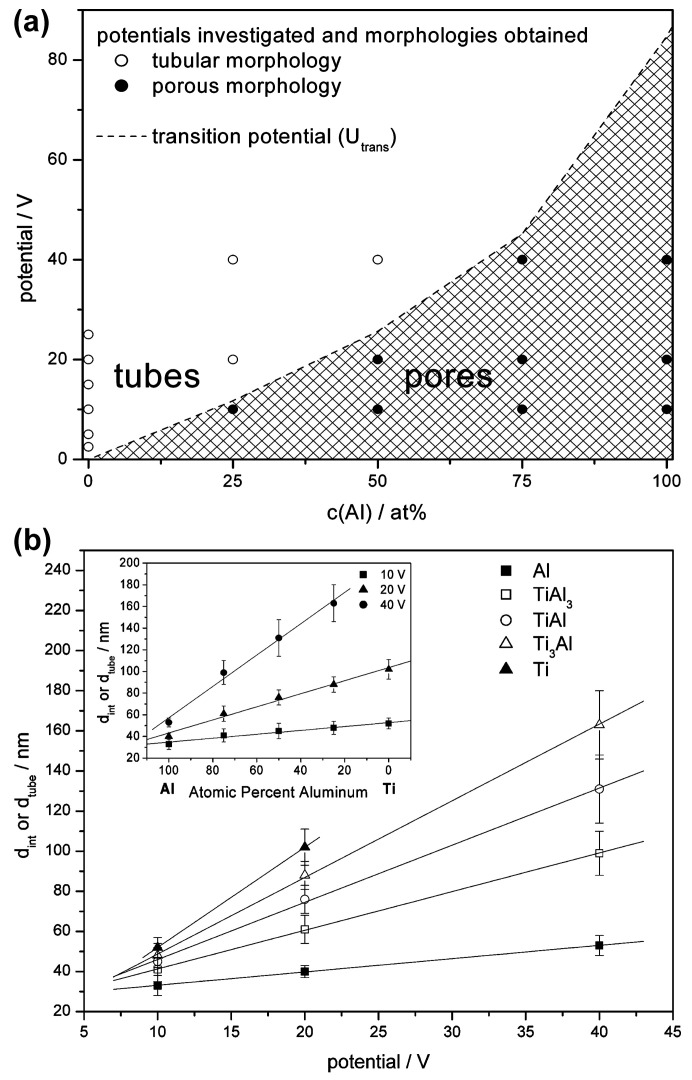
The influence of the chemical composition of substrate material (Al, Ti, and their intermetallics) on the type of morphology (**a**) and interpore/intertube distance (**b**) for effects of anodizing in 1 M H_2_SO_4_ containing 0.15 wt.% HF at various voltages. Reprinted with permission from ACS [65].

A similar, but deepened and fundamental study, was reported by Schmuki’s group: pure Al, TiAl_3_, TiAl, Ti_3_Al, and pure Ti [65]. In order to study the impact of the chemical composition of the substrate on the formation of the nanostructures, the same operating conditions were set up, namely: 1 M H_2_SO_4_ containing 0.15 wt.% HF, and applied voltages of 10, 20, or 40 V (Table 2). The reported study clearly shows an evolution of morphology from the nanopores, formed on pure aluminum, to nanotubes formed on pure titanium (Figure 1). The greater the titanium content in the anodized substrate, the more likely that the nanotubes, instead of the nanopores, are formed. Quantitatively, there is a transition voltage (potential) above which the nanotubes are formed instead of the nanopores. The more Ti in the substrate, the lower this transition voltage (potential) (Figure 2a). For example, when Ti_3_Al is anodized at 10 V, nanopores are obtained, while at higher voltages the nanotubes are formed. But when TiAl_3_ is anodized, the nanopores are formed independently of the voltage.

The chemical composition of the substrate impacts not only the type of morphology (pores, or tubes) of the obtained oxides, but also significantly impacts the morphological features of the nanopores and nanotubes (Figure 2b). Namely, the greater the Ti content in the substrate material, the greater the slope of the interpore/intertube distance vs. anodizing voltage (potential). Also, when the interpore/intertube distance is plotted versus the chemical composition of the alloy (Figure 2b inset), it is apparent that the chemical composition of the substrate directly impacts the morphological features of the oxides: for 40 V intertube distance grown on pure Ti is ca. three times greater than the interpore distance grown on pure Al. Also thickness of the oxide differs, depending on the chemical composition of the substrate. Current density recorded during anodization at 20 V drops from ca. 10 mA/cm^2^ for pure Al to ca. 0.1 mA/cm^2^ for pure Ti. Anodizing is a Faradaic process; thus, the current density has a direct impact on the mass, and consequently, on the thickness of the formed oxides. For pure Al, thickness of the formed oxides was ca. 40 µm, while already for TiAl_3_ it dropped to 0.960 µm. When Ti_3_Al was anodized, the thickness of the formed oxides was as low as 0.290 µm. Anodization of the intermetallic compounds from the Ti-Al phase diagram allowed understanding the evolution from nanopores to nanotubes. It is also worth noting that the more Ti in the substrate, and the greater the anodizing voltage, the greater the oxide growth rate. A greater oxide growth rate causes greater stress, especially in the triple points where the anodic oxide cell boundaries meet. Such great stresses make the oxide more prone to the chemical reaction with the fluorides in the electrolyte, thus water-soluble anions like [TiF_6_]^2−^ are formed. It means the more Ti in the substrate and the greater the anodizing voltage, the more likely nanotubes instead of the nanopores are formed (Figure 2).

## 4. Anodization of Ni_3_Al

**Figure 3:**
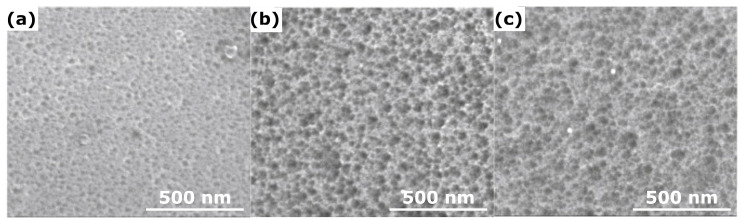
FE-SEM images of nanoporous oxides formed on Ni_3_Al by anodizing in 0.3 M citric acid at 0 °C, at 2 (**a**), 6 (**b**), and 10 V (**c**). Reprinted with permission from Elsevier [68].

Ni_3_Al intermetallic alloys are widely used as a structural material due to their high strength and outstanding mechanical performance at elevated temperatures, due to the formation of a passive oxide layer on the surface [66]. However, the electrochemistry of the Ni_3_Al makes it difficult to anodize: according to the Pourbaix pH range, in the case of Al anodizing, it ranges from ca. 4 to 9, while for Ni it ranges from ca. 9 to 12 [67]. In theory, it makes Ni_3_Al anodizing almost impossible. Typical electrolytes for aluminum anodizing, like sulfuric acid, oxalic acid, or phosphoric acid, fail due to corrosion [68]. The only option was finding a mild, organic acid with a relatively high pH [68,69]. It was found that an anodic oxide can be formed in 0.3 M citric acid at voltages ranging from 2 to 12 V [68,69] (Table 3). However, the morphology of the formed oxide is far from the almost perfect arrangement of AAO nanopores. It is rather a sponge-like nanoporous morphology than an ordered one (Figure 3). The average value of the pore diameter of the formed oxide grows linearly from 18.9 ± 5.0 nm (2.0 V, 0 °C) to 32.0 ± 18.7 nm (anodization at 12.0 V, 30 °C). Due to the sponge-like and non-ordered morphology, one can notice that the standard deviation of the pore diameter is high. In contrast to the anodic alumina grown on pure aluminum, the increase in electrolyte temperature during Ni_3_Al anodizing did not significantly affect the pore diameter. Also, the interpore distance of the grown oxide linearly increases with the anodizing voltage: 0 °C at 2.0 V gives oxide with interpore distance equal to 56.6 ± 1.4 nm, while at 6.0 V, the interpore distance equals 117.5 ± 11.3 nm. It was proven that the temperature of the electrolyte does not influence the interpore distance, as in the case of pure aluminum anodizing.

Formation of the nanoporous oxide on Ni_3_Al differed significantly from the anodic alumina growth on pure aluminum. Despite relatively high current densities recorded during Ni_3_Al anodizing in 0.3 M citric acid (up to over 40 mA/cm^2^ at 30 °C, 12 V) the oxide growth rate was relatively low, ranging from 0.14 (0 °C, 2 V) to 2.29 µm/h (30 °C, 10 V). It means poor current efficiency of the process, or indicates simultaneous corrosive or gas evolution phenomena. To compare, for example, aluminum anodizing in 0.3 M oxalic acid gives current densities reaching the level of 55 mA/cm^2^ (30 °C, 65 V) with an oxide growth rate up to 55.6 µm/h [70].

## 5. Anodization of Fe-Al Intermetallic Alloys

The most researched aluminides in terms of anodizing are materials from the Fe-Al binary phase diagram. Both iron and aluminum can form passive oxides in the same pH range; therefore, it is assumed that both form anodic oxides without major problems, like corrosion. Additionally, there are also individual publications about iron anodizing in ethylene glycol electrolyte containing 0.1 M NH_4_F and 1.5 M H_2_O [71], or 0.5% NH_4_F with 3% H_2_O [72]. In both cases, iron oxides nanotubes were formed. According to the Fe-Al phase diagram, both elements form numerous phases, like Fe_3_Al, FeAl, FeAl_2_, Fe_2_Al_5_, and FeAl_3_, which are interesting due to the fundamentals of the anodic oxides formation [73,74].

**Figure 4:**
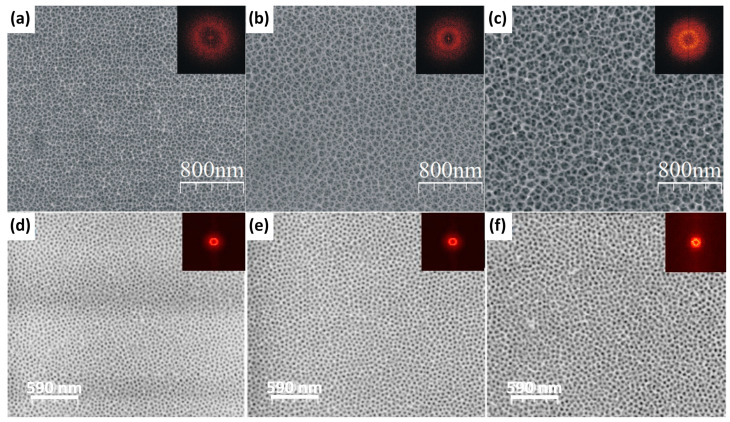
FE-SEM images with their Fast Fourier Transforms (top-right insets) of nanoporous oxides formed on FeAl (**a–c**) [75] and FeAl_3_ [77] in 20% wt. sulfuric acid at 10 (**a**), 15 (**b**), 20 (**c**), 17.5 (**d**), 20 (**e**), and 22.5 V (**f**), respectively. Reprinted with permission from Elsevier [75].

The first attempt was to anodize FeAl [75,76] and FeAl_3_ [77,78] in sulfuric acid, which is a standard approach in aluminum anodizing (Table 4). On first glance, it was noticed already during the materials synthesis that the recorded current densities were much greater than for pure aluminum anodizing. Namely, anodization of FeAl and FeAl_3_ in 20 wt.% sulfuric acid at 0 °C resulted in current densities reaching 10, or 0.7 A/cm^2^ for FeAl [75] and FeAl_3_ [77], respectively, while for pure aluminum anodizing, the registered current densities were at the level of mA/cm^2^ [12]. Thus, it already impacted the oxide growth rate, which was up to ca. 743 µm/h, and ca. 464 µm/h for FeAl [75], and FeAl_3_ [77], respectively. Despite an extremely rapid oxide growth rate, the morphology of the obtained oxides was still nanoporous (Figure 4); however, the slope of the pore diameter versus applied anodizing voltage, or interpore distance vs. anodizing voltage, was much greater than in the case of pure aluminum anodizing. Anodic oxides grow within the field-assisted etching mechanism; thus, for a high oxide growth rate, there is a risk that the balance will be disturbed. In such a situation, the growth of the oxide would be much greater than the secondary etching of the oxide by the attracted electrolyte anions, and there is a risk that no pores could be formed. Fast Fourier Transforms (FFT) of the FE-SEM images confirm poor ordering of the pores: for highly ordered, hexagonally arranged nanoporous alumina, the FFT image is six distinct dots in the corners of a hexagon [13,18,70], while for the disordered structures, the FFT is a blurred ring, what is observed in Figure 4. Simultaneously, ordering of the pores, when compared to pure anodic alumina, was much worse [75,76,77]. For FeAl the oxide was totally disordered (Figure 4a–c), while for FeAl_3,_ anodizing, some hexagonal arrangement of the nanopores can be noticed (Figure 4d–f). It is apparent that the more iron in the substrate material, the worse the ordering of the formed oxide nanopores. This situation is analogous to one with Ti-Al [65], discussed above. Anodization of iron results in the formation of nanotubes [71,72], like in the case of anodization of titanium [43,44,45,46,47,48,49], while anodization of aluminum results in the formation of the hexagonally arranged nanoporous oxide [12,13,14,15,16,17,18,19,20,21]. Solubility of FeSO_4_ is a few times greater than Al_2_(SO_4_)_3_, analogously to the formation of [TiF_6_]^2−^ and [AlF_4_]^−^. Therefore, the more iron in the substrate, and consequently, the more iron in the formed oxide, the easier the etching is, but also the greater the oxide growth rate and the expansion of the oxide. Therefore, poor ordering of the oxide on FeAl is caused due to the operating conditions and substrate composition—the formed disordered oxide is in the “transition zone” between the pores and the nanotubes. Although the oxide formed in FeAl_3_ is ordered because it is much closer to pure aluminum, with its composition.

**Figure 5:**
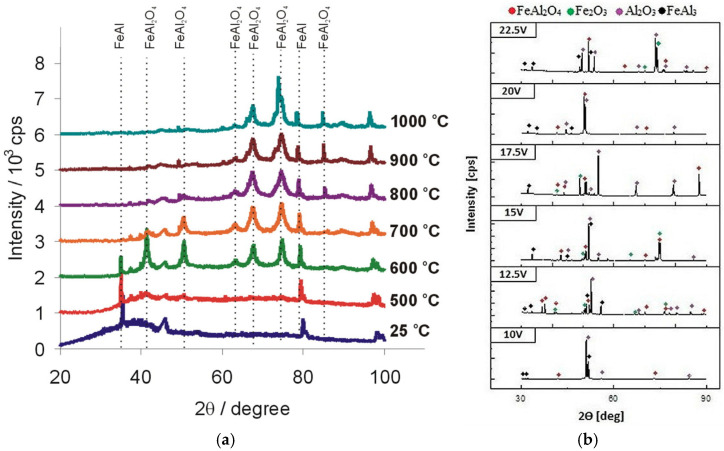
XRD patterns of nanoporous oxides formed on FeAl (**a**) [75] and FeAl_3_ (**b**) [77] in 20% wt. sulfuric acid. Reprinted with permission from Elsevier [75].

The most interesting finding of the research on iron aluminides anodizing in sulfuric acid is the composition of the oxide. It contains not only aluminum and oxygen, but also iron. When FeAl was anodized, the oxide samples were amorphous, but after annealing, a phase transition took place, and crystalline FeAl_2_O_4_ appeared, whose presence confirms that the oxide layer is made of both alumina and iron oxides (Figure 5a) [75]. As can be seen in the X-ray diffraction (XRD) pattern, certain reflections, since 800 °C, decrease or vanish, while others appear since 600 °C, which means that bigger and more stable crystallites with different orientations are formed. As an example, the intensity of reflection at ca. 74.7° can be followed: it appears after annealing at 600 °C and grows with the annealing temperature, reaching its maximum intensity at 1000 °C, dominating other reflections in the pattern.

In the case of FeAl_3_ anodizing, interestingly, the as-obtained samples had crystalline phases, namely Al_2_O_3_, Fe_2_O_3_, and FeAl_2_O_4_, in their structures, which was ascribed to relatively high current densities recorded during anodizing (Figure 5b) [77]. Moreover, the composition of the formed oxides depends on the applied anodizing voltage. Therefore, the properties of the oxide, related to the structure, are strongly related to the applied voltage: it has been shown that the greater the anodizing voltage, the lower the band gap of the formed oxides: for anodized FeAl it dropped from ca. 3.51 eV (5.0 V) to ca. 2.09 eV (17.5 V) [75], while for anodized FeAl_3_ it dropped from ca. 2.5 eV (10.0 V) to 2.1 eV (22.5 V) [78]. Due to the lower content of iron, FeAl_3_ could be anodized at greater voltages than FeAl; however, the drop of band gap with anodizing voltage was not as steep as in the case of FeAl, because less iron oxide was in the nanoporous oxide, which was confirmed directly by chemical composition analyses, as well as by better ordering of the nanopores. The anodically formed mixed alumina–iron oxides, with tunable band gap and highly developed surface area, have great potential to be applied in photocatalysis. Therefore, the first papers in the field can lay a strong foundation for further applied research. The results can lead to the following conclusion: the greater the iron content in the substrate material, the narrower the anodizing voltage range. For both types of materials, the greater the anodizing voltage, the greater the pore diameter and interpore distance that results from the mechanism of the oxide growth. Also, the greater the anodizing voltage, the greater the iron content in the oxide (Fe_2_O_3_ and FeAl_2_O_4_ band gaps are 2.0–2.2 eV, and 2.0–3.5 eV, respectively, while for alumina it equals 4.3 eV), and consequently, the lower the band gap. Therefore, the composition of the oxides, and consequently, the properties, can be steered by the operating conditions.

In pure aluminum anodizing, the electrolyte that allows for oxidizing a metallic substrate at a wider range of voltages than sulfuric acid is oxalic acid [13,70]. Thus, it was a quite natural approach to also test FeAl anodizing in 0.3 M oxalic acid [79]. Moreover, in order to reduce the current densities during the process, analogously to the case of aluminum, a polyhydroxyl alcohol was added to increase the viscosity and decrease the ionic mobility [16]. The recorded current densities were much lower than in the case of FeAl anodizing in sulfuric acid, namely, they were below 0.5 A/cm^2^, thus, the oxide growth rate was much smaller, ranging from 18 (10 V) to 92.4 µm/h (40 V) [79]. Unfortunately, the morphology of the formed oxide was disordered, similar to that formed on Ni_3_Al [68,69].

Del Olmo et al. reported iron-rich, Fe_3_Al anodizing in a tartaric-sulfuric acid electrolyte. Tartaric-sulfuric acid anodizing (TSA) is a common procedure in technical aluminum alloys anodizing for corrosion protection, thus it could promise a positive outcome of the research [7]. Moreover, sulfuric acid was previously proven to be a suitable electrolyte for FeAl and FeAl_3_ alloy anodizing [75,76,77,78]. Additionally, the authors have added ethylene glycol, like in [79], in order to prevent catastrophic current density flow, causing the anode’s “*burning*” (Table 4). Analogously to other iron aluminides, high current densities, reaching a fraction of A/cm^2^, were also recorded here. It has to be noticed that also in this case the voltage window for anodizing is rather narrow—at 20 V, independently of the amount of added ethylene glycol (EG), the samples were burning, thus the desired, nanoporous oxide was obtained only for anodizing at 5 and 10 V. Also in the case of Fe_3_Al, the greater the voltage, the greater the oxide growth rate (from 30 µm/h for 10 V to 126 µm/h for 20 V; no EG). Of course, the more the EG in the electrolyte, the lower the current densities, and consequently, the lower the oxide growth, even as low as 6 and 18 µm/h for 10 and 20 V, respectively, when 50% of EG was in the electrolyte. Furthermore, despite its high-iron alloy, not only typical in Fe-Al alloys, anodizing γ-Al_2_O_3_, α-Fe_2_O_3_, or FeAl_2_O_4_ was detected with XRD. Here also Fe_3_O_4_, FeAlO_3_ were found to be present in the XRD pattern. Also in this case, the obtained nanoporous oxides were semiconductors, and their band gap ranged from 1.91 (50% EG, 15 V) to 2.20 eV (25% EG, 20 V) for as-obtained oxides and from 2.13 (0% EG, 10 V, and 25% EG, 10 V) to 2.30 eV (50% EG, 20 V) for the annealed samples (900 °C).

**Figure 6:**
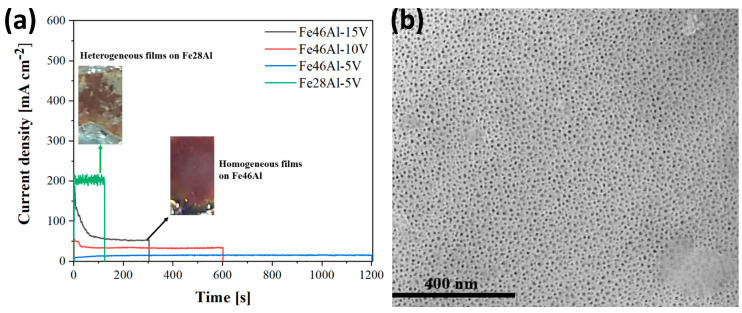
Current density transients of Fe28Al and Fe46Al anodizing (**a**) and top-view FE-SEM image (**b**) of Fe46Al after anodizing at 10 V. Reprinted from [80] (article is licensed under a Creative Commons Attribution 4.0 International License).

Del Olmo et al. also tested on Fe-Al alloys another type of anodizing, quite common in aluminum technical alloys. They used boric acid (0.25 M) with sulfuric acid (1 M) as an electrolyte. The process is commonly known as Boric-Sulfuric Acid Anodizing, BSAA [80]. What is remarkable is that the authors anodized two alloys, Fe28Al, and Fe46Al, and it was found that anodizing Fe28Al alloys does give a very noisy current density transient (Figure 6a), which translates into inhomogeneous layer growth, accompanied by corrosion. Thus, for Fe28Al via BSAA, no homogeneous oxide layers were obtained. It shows how this type of starting material is susceptible to the electrolyte: BSAA does not provide a homogeneous, nanoporous oxide layer for Fe28Al; however, the same material was successfully anodized using the TSA approach [80]. Although BSAA of Fe46Al provided nanoporous, homogeneous oxide (Figure 6a,b), as previously, in this case XRD also reported the formation of α-Fe_2_O_3_, γ-Fe_2_O_3_, Fe_3_O_4_, FeAl_2_O_4_, FeAlO_3_, and γ-Al_2_O_3_. Again, also here, the band gap was within the range of 2.05–2.20 eV and dropped to 2.00–2.10 eV after annealing. Interestingly, the authors chemically sealed the anodic oxides formed on Fe46Al by immersion in an aqueous solution of CuSO_4_, analogously to the chemical sealing of anodic alumina pores. It allowed to form such compounds as CuO and CuFe_2_O_4_ and narrowed the band gap to 1.90 eV.

Another quite commonly applied electrolyte for aluminum anodizing is editronic acid (C_2_H_8_O_7_P_2_) [81]. When typical aluminum anodizing allows formation of the nanoporous layer in the range of 15–25 V (mild anodizing), editronic acid allows application voltages as high as 210 V. For Fe-Al intermetallic alloys, the voltage range has also been widened thanks to the application of this electrolyte. In [82], del Olmo et al. reported anodization of Fe40Al in 0.3 M editronic acid in solutions containing various amounts of ethylene glycol, which allowed application of voltages as high as 400 V, when an ethylene glycol-based electrolyte was applied. The greater the EG content in the electrolyte, the greater its viscosity and consequently the lower the ionic mobility [16]. This allows for decreasing the current densities and, as a result, allows anodizing the alloys at greater voltages, reaching even 400 V. In the reported research, the impact of the preparation of the starting material on the effects of anodizing was researched. Two types of materials, traditionally die-cast and sintered samples, were subjected to anodizing. It was revealed that anodizing of traditionally cast alloys did not allow the formation of a homogeneous, uniform, and compact film. The nanostructured oxide was formed on the material obtained via powder metallurgy (sintering). Although not in all cases, the obtained oxide was nanostructured: anodization in a purely EG-based electrolyte at 400 V allowed formation of the oxide with developed surface area, but without the ordered nanopores; however, such oxide had the highest amount of the incorporated phosphorus species from the electrolyte. This finding confirms the successful incorporation of the anions in the electrolyte during FeAl alloy anodizing, a phenomenon quite common in aluminum anodizing [5]. In such samples, after annealing, the presence of phosphorus was so significant that it formed crystalline phases like FePO_4_, noticeable in the XRD patterns.

**Table 4:** Gathered materials from a Fe-Al system with anodizing protocols and the most significant findings.

Type of Material	Bath Composition	Anodizing Conditions	The Most Prominent Findings	Reference
FeAl: 58.11 at.% of Fe, 41.62 at.% of Al and 0.06 at.% of Zr	20% wt. H_2_SO_4_	5–20 V, 0 °C, 60 s.	One and two-step anodizing, as for aluminum, was tested. High current densities, reaching 10 A/cm^2^, were recorded. Both Al and Fe were oxidized and formed nanoporous oxides.	[75]
			The greater the anodizing voltage, the better the ordering of the oxide nanopores.	[76]
FeAl_3_: 22.47 at% of Fe and 77.53 at%		10.0–22.5 V, 0 °C, 60 s.	Two-step anodizing was applied; relatively good ordering of the pores was attained; pore diameter and interpore distance grow linearly with applied voltage. Fe_2_O_3_, and FeAl_2_O_4_ were detected with XRD.	[77]
			XPS and XRD confirm the presence of the iron oxides in the products of anodizing; the greater the anodizing voltage, the lower the band gap of the formed oxides.	[78]
FeAl: 58.11 at.% of Fe, 41.62 at.% of Al and 0.06 at.% of Zr	0.3 M (COOH)_2_ with 20% vol. Ethylene glycol	10–25, and 40 V, 0 °C, 300 s.	Adding ethylene glycol to oxalic acid allowed to decrease the oxide growth rate to decrease; however, the morphology of the formed nanoporous oxides was poorly organized.	[79]
Fe_3_Al; wt.%: 28.0 Al, 5.0 Cr, 0.08 Zr, 0.04 B, and Fe balance	0.5 M H_2_SO_4_ + 1 M tartaric acid, 0, 25, or 50% of ethylene glycol	10, 15, or 20 V, 10 °C, time varied in order to attain the same charge in the circuit	Addition of ethylene glycol (EG) slowed down the oxide growth rate; even sub-10 nm nanoporous oxides were formed; the formed oxides, after annealing (900 °C), were composed of: α-Fe_2_O_3_, Fe_3_O_4_, FeAl_2_O_4_, FeAlO_3_, and γ-Al_2_O_3_. Depending on the anodizing conditions, the band gap of the formed oxides ranged from 1.91 eV to 2.30 eV.	[83]
	0.25 M boric acid + 1 M H_2_SO_4_	5, 10, 15 V, 10 °C,	No stable, homogeneous layer on Fe28Al was obtained.	[80]
Fe46Al; wt.%: 46 Al, 0.04 B, 0.05 Zr, and Fe balance			The nanoporous oxides were sealed with CuSO_4_. Before sealing, the band gap ranged from 2.00 to 2.10 eV (after annealing), and after the sealing, it was 1.90 eV (after annealing).	
Fe40Al; wt.%: 40 Al, 0.08 Zr, 0.04 B, and Fe balance	0.3 M editronic acid; various amounts of ethylene glycol: 0, 50, 75, and 100 vol. %	10–400 V, 25 °C, 1 or 4 h	Traditionally, die-cast and sintered materials were subjected to anodizing. The as-cast alloys were difficult to anodize—inhomogeneous oxides were formed; materials formed via sintering were found to be much better for anodizing. Phosphorous species were incorporated into the grown oxides; the band gap of the formed oxides ranged from 2.45 to 3.00 eV.	[82]
	0.3 M editronic acid with EG, 3:1 vol. ratio	75 V, 20 °C, 2.5 h	Material obtained by the powder metallurgy process was anodized. Nanotubes were formed. Anodizing was followed by annealing and chemical etching. It allowed the obtaining of material with a developed surface area and relatively high photocurrents recorded in 1.0 M NaOH under illumination.	[84]

In continued research, optimized experimental conditions of anodizing on the sintered material were applied: the Fe40Al was anodized in 0.3 M editronic acid with EG, in a 3:1 vol ratio at 75 V for 2.5 h, and as a result, uniform oxide nanotubes were obtained. The anodization was also followed by annealing in the air atmosphere (400 °C, 2 h) and chemical etching (1 M NaOH, 1 h). It is crucial to note that annealing allowed the nanotubular morphology to be kept; however, chemical etching in 1.0 M NaOH due to the reaction with the alumina destroyed the nanotubes, but the surface area of the material was still strongly developed. As a final result, material with a highly developed surface area composed mainly of the iron compounds, covered by a thin layer of alumina was obtained. An electrochemical study using a sun simulator and 1.0 M NaOH allowed to show that the obtained photocurrents were interesting from the point of view of photocatalytic applications of the obtained material.

## 6. Summary

Anodizing is becoming a very popular method for the nanostructured oxide fabrication. Up to now, dozens of various metals have been anodized, and nanoporous, or nanotubular morphologies have been obtained. Anodization of metal aluminides, including gold aluminide, titanium aluminides, nickel aluminide, and iron aluminides, opened a new door for complex nanomaterials formation. Using a facile method, anodizing, complex nanomaterials with a highly developed surface area can be obtained. This research is at its beginning. Firstly, the mechanism of the simultaneous oxidation of both elements has to be fully understood, using in situ methods with a full balance of the composition of the formed materials and changes in the anodizing electrolytes. Secondly, despite having information about the physical properties of the oxides, especially formed on materials from the Fe-Al binary phase diagram, no significant applications have been developed yet. Nevertheless, this new route in the anodizing research may lead to numerous breakthroughs where mixed oxides with a developed surface area are demanded.

## Figures and Tables

**Table 1:** Anodizing protocol and the most significant findings for purple gold, AuAl_2_.

Type of Material	Bath Composition	Anodizing Conditions	The Most Prominent Findings	Reference
AuAl_2_	0.3 M oxalic acid (COOH)_2_	5 or 20 V, 30 or 90 min.	Anodization of purple gold allowed the formation of gold nanoparticles (ca. 30 nm in diameter); application: detection of pyridine (Surface-Enhanced Raman Spectroscopy)	[63]

**Table 2:** Anodizing protocol and the most significant findings for Ti-Al alloys.

Type of Material	Bath Composition	Anodizing Conditions	The Most Prominent Findings	Reference
Al, TiAl_3_, TiAl, Ti_3_Al, Ti	1 M H_2_SO_4_ with 0.15 wt.% HF	10, 20, or 40 V, 30 or 90 min.	Depending on the Ti:Al ratio and anodizing voltage, a nanoporous or nanotubular oxide is formed.	[65]

**Table 3:** Anodizing protocol and the most significant findings for Ni-Al alloys.

Type of Material	Bath Composition	Anodizing Conditions	The Most Prominent Findings	Reference
Ni_3_Al	0.3 M citric acid	2–12 V, 0 or 30 °C, 12 h	The formed oxide was poorly ordered; pore diameter and interpore distance increased linearly with anodizing voltage.	[68]
			An attempt to quantitatively study the ordering of the formed pores	[69]

## Data Availability

No new data were created or analyzed in this study.
